# Redetermination of diaqua­tetra­kis­(dimethyl­formamide-κ*O*)magnesium dichloride

**DOI:** 10.1107/S1600536811027073

**Published:** 2011-07-23

**Authors:** Guido J. Reiss, Ishtvan Boldog, Christoph Janiak

**Affiliations:** a Institut für Anorganische Chemie und Strukturchemie, Heinrich Heine Universität Düsseldorf, Universitätsstrasse 1, D-40225 Düsseldorf, Germany

## Abstract

The crystal structure of the title compound, [Mg(C_3_H_7_NO)_4_(H_2_O)_2_]Cl_2_, in which the Mg ion lies on a crystallographic inversion centre, confirms that of the previous room-temperature study [Pavanello *et al.* (1995 ▶). *Main Group Met. Chem.* **18**, 9–19]. This redetermination at 113 K has improved geometry precision by almost an order of magnitude [e.g. Mg—O(w) (w = water) distances = 2.094 (4) and 2.0899 (7) Å in the old and new structures, respectively] and allowed the water H atoms to be located and their positions refined. In the crystal, O—H⋯Cl hydrogen bonds between the two aqua ligands of the complex mol­ecule and neighboring chloride counter-anions generate supra­molecular chains propagating along [010]. The dicationic [Mg(DMF)_4_(H_2_O)_2_] unit (DMF is dimethyl­formamide) adopts a slightly distorted octa­hedral geometry in which the Mg atom is coordinated by four DMF O atoms in a pseudo-tetra­gonal arrangement and two *trans* aqua ligands.

## Related literature

For the previous structure determination, see: Pavanello *et al.* (1995 ▶). For related structures, see: Lebioda & Lewiński (1980 ▶); Castro *et al.* (2010 ▶). For discussion of hydrogen bonds, see: Etter *et al.* (1990 ▶); Janiak *et al.* (1996 ▶). Dorn *et al.* (2005 ▶); Aakeröy *et al.* (2010 ▶). 
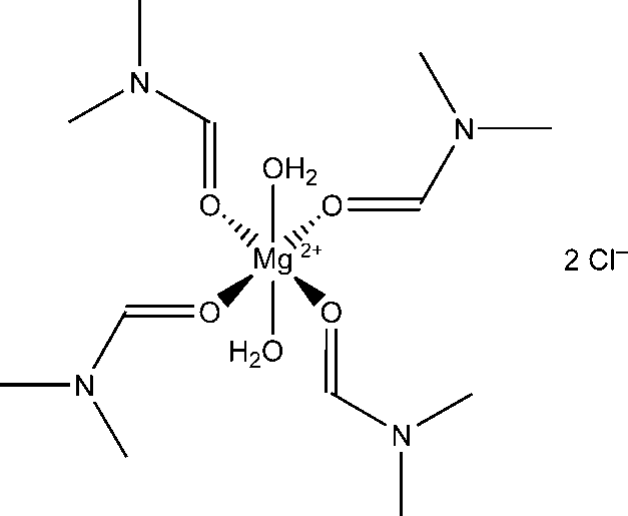


## Experimental

### 

#### Crystal data

[Mg(C_3_H_7_NO)_4_(H_2_O)_2_]Cl_2_
*M* *_r_* = 423.62
Triclinic, 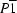

*a* = 8.0284 (3) Å
*b* = 8.0748 (3) Å
*c* = 8.8373 (4) Å
α = 90.803 (3)°
β = 91.330 (3)°
γ = 111.563 (4)°
*V* = 532.51 (4) Å^3^
*Z* = 1
Mo *K*α radiation
μ = 0.37 mm^−1^
*T* = 113 K
0.40 × 0.25 × 0.10 mm

#### Data collection

Oxford Diffraction Xcalibur Eos diffractometer
Absorption correction: multi-scan (*CrysAlis PRO*; Oxford Diffraction, 2010 ▶) *T*_min_ = 0.683, *T*_max_ = 1.000
8374 measured reflections
2443 independent reflections
2381 reflections with *I* > 2σ(*I*)
*R*_int_ = 0.017
3 standard frames every 30 min  intensity decay: none

#### Refinement

*R*[*F* ^2^ > 2σ(*F* ^2^)] = 0.021
*wR*(*F* ^2^) = 0.048
*S* = 1.07
2443 reflections
136 parameters
H atoms treated by a mixture of independent and constrained refinement
Δρ_max_ = 0.29 e Å^−3^
Δρ_min_ = −0.18 e Å^−3^

### 

Data collection: *CrysAlis PRO* (Oxford Diffraction, 2010 ▶); cell refinement: *CrysAlis PRO*; data reduction: *CrysAlis PRO*; program(s) used to solve structure: *SHELXS97* (Sheldrick, 2008 ▶); program(s) used to refine structure: *SHELXL97* (Sheldrick, 2008 ▶); molecular graphics: *DIAMOND* (Crystal Impact, 2009 ▶); software used to prepare material for publication: *publCIF* (Westrip, 2010 ▶).

## Supplementary Material

Crystal structure: contains datablock(s) I, global. DOI: 10.1107/S1600536811027073/hb5919sup1.cif

Structure factors: contains datablock(s) I. DOI: 10.1107/S1600536811027073/hb5919Isup2.hkl

Additional supplementary materials:  crystallographic information; 3D view; checkCIF report

## Figures and Tables

**Table 1:** Selected bond lengths (Å)

Mg—O1	2.0221 (6)
Mg—O2	2.0839 (7)
Mg—O3	2.0899 (7)

**Table 2:** Hydrogen-bond geometry (Å, °)

*D*—H⋯*A*	*D*—H	H⋯*A*	*D*⋯*A*	*D*—H⋯*A*
O3—H31⋯Cl	0.810 (16)	2.348 (16)	3.1528 (8)	172.6 (14)
O3—H32⋯Cl^i^	0.817 (17)	2.326 (18)	3.1408 (8)	175.4 (15)

Symmetry code: (i) 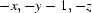.

**Fig. 1.**
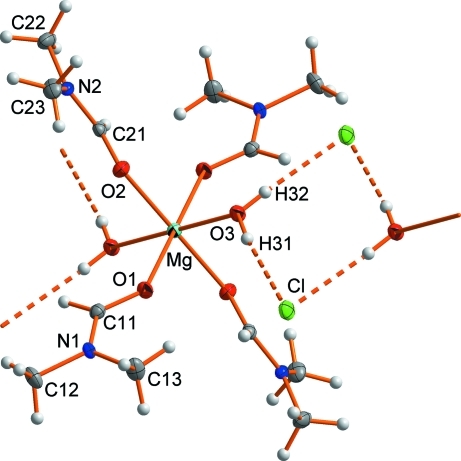
: Molecular structure of [Mg(H2O)2{(CH3)2NCHO}4]Cl2. Hydrogen bonds are indicated as dashed lines (see Table for bond distances and angles). Displacement ellipsoids are drawn at the 60% probability level; H atoms as spheres of arbitrary radii.

## supplementary crystallographic information

### Comment

In the structure of the title compound the complex cation *trans*-[Mg(H_2_O)_2_(DMF)_4_]^2+^ (DMF = HCON(CH_3_)_2_) is located on a center of symmetry. Two DMF ligands show significantly shorter Mg—O bond lengths with 2.0221 (6) Å than the two other DMF ligands and the two aqua ligands (2.0839 (7), 2.0899 (7) Å, respectively).

This distortion is in accord with the reported room temperature structure of the title compound [2.021 (5), 2.094 (5) and 2.094 (4) Å, respectively] (Pavanello *et al.*, 1995) and the structure of [MgK_2_(croconate violet)(H_2_O)_4_] where one of the two Mg—O(croconate) bond distances is significantly longer (2.128 Å) than the other one or the Mg—O(H_2_O) bond distance (2.072 (1), 2.053 (2) Å, respectively) (Castro *et al.*, 2010). Whereas the structure of [Mg(H_2_O)_2_{OC(NH_2_)_2_}_2_]Br_2_ has similar Mg—O(urea) bond distances of 2.050 (1) and 2.078 (1) Å and a longer Mg—O(H_2_O) contact (2.108 (2) Å) (Lebioda & Lewiński, 1980).

Hydrogen bonding as a primary interaction in crytal engineering and supramolecular chemistry is of continous interest (Aakeröy *et al.*, 2010). The hydrogen bonding between the aqua ligands and the chloride counter anions is in the typical range (Dorn *et al.*, 2005; Janiak *et al.*, 1996). The cyclic hydrogen bond motif (Fig.1) which is formed by two aqua ligands of neigboring complexes and two chloride anions features the well known *R*_2_^4^(8) motif (Etter *et al.*,  1990).

Fig. 1 shows the molecular structure with the hydrogen bonding from the aqua ligands to the chloride ions which leads to the supramolecular chain.

### Experimental

Synthesis

42 mg (0.44 mmol) of anhydrous MgCl_2_ (Merck, >98%) and 500 µL of DMF (ACS, H_2_O content max. 0.1%) in a 1.5 ml vial were shaken at r.t. until complete dissolution of the solid. 16 mL of H_2_O (0.88 mmol) were added to the formed solution at once, the solution was homogenized and the vial was placed in an oven preheated at 70°C. After one day the vial was cooled down to r.t. with a rate of 2 K/h to yield large and thick plates of perfect optical quality with dimensions significantly outreaching the millimeter scale. Rapid cooling in an alternative experiment resulted in complete crystallization within one hour. Yet, the formed crystals were of lower quality and their opaqueness indicated some solvent occlusion.The crystalline product redissolves readily in the mother liquor under heating.

The isolated crystals deliquesce quickly in air, whereby hindering the exact determination of the yield. A repeated experiment was performed and the crystals were separated by decanting-off the mother solution, washing the residue with 3 × 1ml of diethyl ether and drying it in an argon stream during a few minutes thus yielding 140 mg (75%) of product.

For IR (ATR) measurements a few transparent crystals were separated directly from the mother solution, dried on a filter paper, ground and measured immediately allowing less then one minute contact with air. IR (ATR): ν (cm^-1^) = 3226(s, br, sh), 2933(*m*), 1649(*vs*), 1501(*m*), 1445(*m*), 1433(*m*),1417(*m*), 1394(*s*), 1252(*m*), 1116(*s*), 1063(*m*),867(w), 679(*s*). The ether washed product had the same spectrum but with an additional weak line at 805 cm^-1^.

### Refinement

A single-crystal suitable for structure determination was harvested from the mother liquor and was directly transferred into the cooling stream of an Oxford-Xcalibur diffractometer equipped with an EOS-CCD detector at 113 K. In the final stages of the refinement the anisotropic displacement parameters of all non-hydrogen atoms were refined.

The hydrogen atoms of the the C—H group of the DMF ligand and the hydrogen atoms of the water ligand were refined freely with individual *U*_iso_ values. The hydrogen atoms of the methyl groups were introduced using a riding model (*SHELXL*; AFIX 137).

### Crystal data

**Table:**

[Mg(C_3_H_7_NO)_4_(H_2_O)_2_]Cl_2_	*Z* = 1
*M**_r_* = 423.62	*F*(000) = 226
Triclinic, *P*1	*D*_x_ = 1.321 Mg m^−^^3^
Hall symbol: -P 1	Mo *K*α radiation, λ = 0.71073 Å
*a* = 8.0284 (3) Å	Cell parameters from 9519 reflections
*b* = 8.0748 (3) Å	θ = 3.1–31.8°
*c* = 8.8373 (4) Å	µ = 0.37 mm^−^^1^
α = 90.803 (3)°	*T* = 113 K
β = 91.330 (3)°	Plate, colourless
γ = 111.563 (4)°	0.40 × 0.25 × 0.10 mm
*V* = 532.51 (4) Å^3^	

### Data collection

**Table:**

Oxford Diffraction Xcalibur Eos diffractometer	2381 reflections with *I* > 2σ(*I*)
Radiation source: fine-focus sealed tube	*R*_int_ = 0.017
graphite	θ_max_ = 27.5°, θ_min_ = 5.1°
ω scans	*h* = −10→10
Absorption correction: multi-scan (*CrysAlis PRO*; Oxford Diffraction, 2010)	*k* = −10→10
*T*_min_ = 0.683, *T*_max_ = 1.000	*l* = −11→11
8374 measured reflections	3 standard reflections every 30 min
2443 independent reflections	intensity decay: none

### Refinement

**Table:**

Refinement on *F*^2^	Secondary atom site location: difference Fourier map
Least-squares matrix: full	Hydrogen site location: inferred from neighbouring sites
*R*[*F*^2^ > 2σ(*F*^2^)] = 0.021	H atoms treated by a mixture of independent and constrained refinement
*wR*(*F*^2^) = 0.048	*w* = 1/[σ^2^(*F*_o_^2^) + (0.01*P*)^2^ + 0.25*P*] where *P* = (*F*_o_^2^ + 2*F*_c_^2^)/3
*S* = 1.07	(Δ/σ)_max_ < 0.001
2443 reflections	Δρ_max_ = 0.29 e Å^−^^3^
136 parameters	Δρ_min_ = −0.18 e Å^−^^3^
0 restraints	Extinction correction: *SHELXL97* (Sheldrick, 2008), Fc^*^=kFc[1+0.001xFc^2^λ^3^/sin(2θ)]^-1/4^
Primary atom site location: structure-invariant direct methods	Extinction coefficient: 0.058 (3)

### Special details

**Table:**

Experimental. CrysAlisPro, Oxford Diffraction Ltd., Version 1.171.34.44 Empirical absorption correction using spherical harmonics, implemented in SCALE3 ABSPACK scaling algorithm (Oxford Diffraction, 2010).
Geometry. All e.s.d.'s (except the e.s.d. in the dihedral angle between two l.s. planes) are estimated using the full covariance matrix. The cell e.s.d.'s are taken into account individually in the estimation of e.s.d.'s in distances, angles and torsion angles; correlations between e.s.d.'s in cell parameters are only used when they are defined by crystal symmetry. An approximate (isotropic) treatment of cell e.s.d.'s is used for estimating e.s.d.'s involving l.s. planes.
Refinement. Refinement of *F*^2^ against ALL reflections. The weighted *R*-factor *wR* and goodness of fit *S* are based on *F*^2^, conventional *R*-factors *R* are based on *F*, with *F* set to zero for negative *F*^2^. The threshold expression of *F*^2^ > σ(*F*^2^) is used only for calculating *R*-factors(gt) *etc*. and is not relevant to the choice of reflections for refinement. *R*-factors based on *F*^2^ are statistically about twice as large as those based on *F*, and *R*- factors based on ALL data will be even larger.

### Fractional atomic coordinates and isotropic or equivalent isotropic displacement parameters (Å^2^)

**Table:**

	*x*	*y*	*z*	*U*_iso_*/*U*_eq_	
Mg	0.0000	0.0000	0.0000	0.00919 (10)	
Cl	0.24638 (3)	−0.39591 (3)	0.17847 (3)	0.01591 (8)	
O1	0.24626 (9)	0.07858 (9)	0.10106 (8)	0.01376 (14)	
C11	0.37264 (12)	0.22376 (12)	0.12339 (10)	0.01210 (18)	
H11	0.3638 (16)	0.3329 (16)	0.0934 (13)	0.014 (3)*	
N1	0.52502 (10)	0.23979 (10)	0.19295 (9)	0.01220 (16)	
C12	0.66252 (13)	0.41472 (13)	0.22648 (12)	0.0181 (2)	
H121	0.6217	0.5055	0.1910	0.027*	
H122	0.7705	0.4241	0.1767	0.027*	
H123	0.6858	0.4296	0.3338	0.027*	
C13	0.56012 (13)	0.08489 (13)	0.24367 (13)	0.0190 (2)	
H131	0.4636	−0.0216	0.2097	0.029*	
H132	0.5696	0.0877	0.3522	0.029*	
H133	0.6702	0.0862	0.2026	0.029*	
O2	0.11438 (9)	0.09777 (9)	−0.20529 (7)	0.01321 (14)	
C21	0.04046 (12)	0.16098 (12)	−0.30109 (10)	0.01128 (18)	
H21	−0.0740 (16)	0.1740 (15)	−0.2843 (13)	0.012 (3)*	
N2	0.10760 (11)	0.21837 (10)	−0.43429 (9)	0.01286 (17)	
C22	0.00617 (15)	0.27866 (13)	−0.54511 (11)	0.0188 (2)	
H221	−0.0977	0.2868	−0.4989	0.028*	
H222	−0.0306	0.1953	−0.6292	0.028*	
H223	0.0801	0.3936	−0.5802	0.028*	
C23	0.28056 (14)	0.21630 (14)	−0.47797 (12)	0.0186 (2)	
H231	0.3473	0.2065	−0.3893	0.028*	
H232	0.3459	0.3246	−0.5282	0.028*	
H233	0.2625	0.1164	−0.5453	0.028*	
O3	0.02957 (10)	−0.24245 (9)	−0.04443 (8)	0.01385 (15)	
H31	0.094 (2)	−0.2735 (19)	0.0112 (17)	0.030 (4)*	
H32	−0.046 (2)	−0.333 (2)	−0.0815 (19)	0.039 (4)*	

### Atomic displacement parameters (Å^2^)

**Table:**

	*U*^11^	*U*^22^	*U*^33^	*U*^12^	*U*^13^	*U*^23^
Mg	0.0095 (2)	0.0089 (2)	0.0092 (2)	0.00346 (16)	−0.00043 (15)	0.00063 (15)
Cl	0.01812 (12)	0.01091 (11)	0.01905 (13)	0.00586 (9)	−0.00115 (8)	−0.00009 (8)
O1	0.0110 (3)	0.0130 (3)	0.0159 (3)	0.0029 (3)	−0.0024 (2)	0.0007 (3)
C11	0.0140 (4)	0.0124 (4)	0.0107 (4)	0.0057 (4)	0.0016 (3)	0.0010 (3)
N1	0.0102 (4)	0.0101 (4)	0.0154 (4)	0.0026 (3)	0.0004 (3)	−0.0009 (3)
C12	0.0130 (4)	0.0135 (4)	0.0240 (5)	0.0008 (4)	0.0002 (4)	−0.0050 (4)
C13	0.0130 (4)	0.0159 (5)	0.0294 (6)	0.0071 (4)	−0.0036 (4)	0.0018 (4)
O2	0.0143 (3)	0.0143 (3)	0.0119 (3)	0.0063 (3)	0.0014 (2)	0.0027 (2)
C21	0.0127 (4)	0.0081 (4)	0.0122 (4)	0.0030 (3)	0.0005 (3)	−0.0013 (3)
N2	0.0161 (4)	0.0115 (4)	0.0108 (4)	0.0048 (3)	0.0011 (3)	0.0006 (3)
C22	0.0289 (5)	0.0159 (5)	0.0131 (5)	0.0099 (4)	−0.0020 (4)	0.0022 (4)
C23	0.0185 (5)	0.0189 (5)	0.0171 (5)	0.0050 (4)	0.0074 (4)	0.0003 (4)
O3	0.0161 (3)	0.0106 (3)	0.0154 (3)	0.0058 (3)	−0.0029 (3)	−0.0009 (3)

### Geometric parameters (Å, °)

**Table:**

Mg—O1^i^	2.0221 (6)	C13—H132	0.9600
Mg—O1	2.0221 (6)	C13—H133	0.9600
Mg—O2	2.0839 (7)	O2—C21	1.2413 (11)
Mg—O2^i^	2.0839 (7)	C21—N2	1.3237 (12)
Mg—O3^i^	2.0899 (7)	C21—H21	0.977 (12)
Mg—O3	2.0899 (7)	N2—C23	1.4557 (12)
O1—C11	1.2461 (11)	N2—C22	1.4584 (12)
C11—N1	1.3182 (12)	C22—H221	0.9600
C11—H11	0.951 (12)	C22—H222	0.9600
N1—C13	1.4539 (12)	C22—H223	0.9600
N1—C12	1.4589 (12)	C23—H231	0.9600
C12—H121	0.9600	C23—H232	0.9600
C12—H122	0.9600	C23—H233	0.9600
C12—H123	0.9600	O3—H31	0.810 (16)
C13—H131	0.9600	O3—H32	0.817 (17)
			
O1^i^—Mg—O1	180.00 (2)	H122—C12—H123	109.5
O1^i^—Mg—O2	89.71 (3)	N1—C13—H131	109.5
O1—Mg—O2	90.29 (3)	N1—C13—H132	109.5
O1^i^—Mg—O2^i^	90.29 (3)	H131—C13—H132	109.5
O1—Mg—O2^i^	89.71 (3)	N1—C13—H133	109.5
O2—Mg—O2^i^	180.00 (6)	H131—C13—H133	109.5
O1^i^—Mg—O3^i^	86.14 (3)	H132—C13—H133	109.5
O1—Mg—O3^i^	93.86 (3)	C21—O2—Mg	123.01 (6)
O2—Mg—O3^i^	89.34 (3)	O2—C21—N2	124.11 (9)
O2^i^—Mg—O3^i^	90.66 (3)	O2—C21—H21	122.6 (7)
O1^i^—Mg—O3	93.86 (3)	N2—C21—H21	113.3 (7)
O1—Mg—O3	86.14 (3)	C21—N2—C23	121.50 (8)
O2—Mg—O3	90.66 (3)	C21—N2—C22	120.72 (8)
O2^i^—Mg—O3	89.34 (3)	C23—N2—C22	117.73 (8)
O3^i^—Mg—O3	180.00 (4)	N2—C22—H221	109.5
C11—O1—Mg	135.31 (6)	N2—C22—H222	109.5
O1—C11—N1	123.58 (9)	H221—C22—H222	109.5
O1—C11—H11	121.5 (7)	N2—C22—H223	109.5
N1—C11—H11	114.9 (7)	H221—C22—H223	109.5
C11—N1—C13	121.47 (8)	H222—C22—H223	109.5
C11—N1—C12	121.02 (8)	N2—C23—H231	109.5
C13—N1—C12	117.48 (8)	N2—C23—H232	109.5
N1—C12—H121	109.5	H231—C23—H232	109.5
N1—C12—H122	109.5	N2—C23—H233	109.5
H121—C12—H122	109.5	H231—C23—H233	109.5
N1—C12—H123	109.5	H232—C23—H233	109.5
H121—C12—H123	109.5	H31—O3—H32	106.7 (15)

Symmetry codes: (i) −*x*, −*y*, −*z*.

### Hydrogen-bond geometry (Å, °)

**Table:**

*D*—H···*A*	*D*—H	H···*A*	*D*···*A*	*D*—H···*A*
O3—H31···Cl	0.810 (16)	2.348 (16)	3.1528 (8)	172.6 (14)
O3—H32···Cl^ii^	0.817 (17)	2.326 (18)	3.1408 (8)	175.4 (15)

Symmetry codes: (ii) −*x*, −*y*−1, −*z*.
